# The Growing Impact of Severe Obesity on 90-Day Outcomes After Elective Primary Total Hip Arthroplasty: A National Propensity-Matched Study

**DOI:** 10.1016/j.artd.2026.102008

**Published:** 2026-04-09

**Authors:** David Maman, Yaniv Steinfeld, Yaron Berkovich

**Affiliations:** a Department of Orthopaedic Surgery, Carmel Medical Center, Haifa, Israel; b Technion – Israel Institute of Technology, The Ruth and Bruce Rappaport Faculty of Medicine, Technion City, Haifa, Israel

**Keywords:** Total hip arthroplasty, Severe obesity, Morbid obesity, Readmission, Revision surgery, Reoperation

## Abstract

**Background:**

Severe obesity is increasingly prevalent among patients undergoing total hip arthroplasty (THA), yet contemporary national data evaluating its impact on early complications and revision risk remain limited. We evaluated temporal trends and 90-day outcomes associated with severe obesity after elective primary THA.

**Methods:**

We performed a retrospective cohort study using the Nationwide Readmissions Database (2020-2022). Elective primary THAs performed on hospital day 0 were identified. Severe obesity was defined by ICD-10-CM codes E66.01 or E66.2. To ensure complete follow-up, 90-day analyses included procedures performed between January and September. Propensity score matching (1:1, nearest neighbor without replacement) adjusted for demographics, payer, hospital characteristics, and major comorbidities. The primary outcome was all-cause 90-day readmission. Secondary outcomes included reoperation, true component-level revision, and resource utilization.

**Results:**

Among 366,374 weighted elective THAs, 28,798 (7.9%) involved severe obesity. Prevalence increased from 7.4% in 2020 to 8.7% in 2022 (*P* < .001). After matching (55,976 patients), severe obesity was associated with higher rates of acute kidney injury (3.8% vs 2.0%) and blood loss anemia (17.2% vs 12.8%) (both *P* < .001), longer length of stay, and greater charges. Ninety-day readmission occurred in 6.7% vs 5.8% (Odds Ratio [OR]: 1.32; 95% Confidence Intervals [CI]: 1.23-1.41). Severe obesity was associated with higher odds of any procedure (OR: 1.45), hip-related reoperation (OR: 1.57), and component-level revision (OR: 1.53) (all *P* < .001).

**Conclusions:**

Severe obesity is increasingly prevalent and independently associated with higher early complications, readmission, reoperation, and revision following elective primary THA.

**Level of Evidence:**

Level III.

## Introduction

Total hip arthroplasty (THA) is one of the most successful and frequently performed procedures in orthopaedic surgery, with continued expansion driven by aging populations and increasing osteoarthritis prevalence [1,2]. In parallel, obesity has emerged as one of the most prevalent and clinically consequential comorbidities in contemporary arthroplasty practice [3]. National epidemiologic data demonstrate a steady rise in obesity and severe obesity across the United States, directly influencing the demographic profile of patients undergoing elective THA [3,4].

Importantly, the contemporary arthroplasty landscape has undergone substantial transformation following the COVID-19 pandemic. Elective arthroplasty volumes, patient selection patterns, outpatient migration, and perioperative care pathways have evolved rapidly during this period. As a result, earlier studies—largely derived from pre-2020 cohorts—may not fully reflect current practice patterns or risk profiles. [5,6] In parallel, there has been a progressive shift toward anterior and laterally based surgical approaches for THA, which may be more technically challenging in patients with severe obesity due to soft tissue envelope and exposure constraints. Whether severe obesity continues to confer elevated early complication and revision risk in this modern, postpandemic era of evolving surgical techniques and enhanced recovery pathways remains incompletely defined.

Obesity has been associated with increased perioperative morbidity following THA, including higher rates of wound complications, infection, prolonged length of stay (LOS), and increased cost of care [[5], [6], [7], [8]]. Recent large database and registry studies have demonstrated that severely obese and super-obese patients may experience disproportionately elevated risks of periprosthetic joint infection (PJI), readmission, and early complications compared with nonobese counterparts [6,9]. However, prior database studies predominantly evaluate readmission or general complications without distinguishing clinically meaningful surgical escalation—specifically true component-level revision within the early postoperative window. This distinction is critical, as early revision represents definitive implant-related failure rather than transient postoperative morbidity.

Contemporary national analyses that simultaneously examine post-COVID trends, modern care pathways, and true early revision risk in severely obese patients remain limited. Early failure after THA commonly manifests within the first 90 days, particularly through infection- and wound-related pathways that may necessitate reoperation or component exchange [6,9]. Understanding how severe obesity influences not only readmission but also the precise procedural burden and etiologic drivers of early revision has direct implications for preoperative risk stratification, perioperative optimization, and bundled-payment models [8,10,11].

Therefore, the purposes of this study were to (1) evaluate contemporary temporal trends in severe obesity among patients undergoing elective primary THA in the United States from 2020 to 2022, and (2) quantify the association between severe obesity and 90-day outcomes—including all-cause readmission, hip-related reoperation, true component-level revision, and resource utilization—using a national propensity score-matched cohort. We hypothesized that severe obesity would be increasingly prevalent and independently associated with higher odds of early surgical escalation within 90 days following elective primary THA.

Accordingly, this study addresses a key contemporary knowledge gap by providing a national analysis of severe obesity in the post-COVID-19 arthroplasty era with complete 90-day follow-up, while uniquely distinguishing true component-level revision from general readmission events. This approach enables evaluation of clinically meaningful early surgical escalation in the context of modern perioperative pathways and evolving surgical techniques, which have not been fully captured in earlier prepandemic registry studies.

## Material and methods

### Data source and study design

We performed a retrospective cohort study using the Nationwide Readmissions Database (NRD) for years 2020-2022. The NRD is an all-payer U.S. administrative database developed as part of the Healthcare Cost and Utilization Project and enables analysis of hospitalizations and readmissions within the same calendar year using verified patient linkage identifiers. Discharge-level sampling weights were applied to generate national estimates. Because the NRD contains deidentified data, this study was exempt from institutional review board approval and informed consent.

### Cohort identification

We initially identified 919,501 hospitalizations in which THA was recorded as the index procedure during the study years. Elective primary THA admissions were identified using ICD-10-PCS procedure coding and restricted to routine inpatient elective admissions.

Severe obesity was defined using ICD-10-CM diagnosis codes E66.01 and E66.2, corresponding broadly to body mass index, BMI ≥35 kg/m^2^ (class II-III obesity, including morbid and super obesity). Patients not meeting these criteria were categorized as “non-severely obese,” a heterogeneous group including normal weight, overweight, and class I obese individuals. Therefore, comparisons in this study reflect the incremental risk attributable to severe obesity rather than obesity in general.

### Exclusion criteria and follow-up window

To establish a uniform elective primary THA cohort appropriate for 90-day outcomes, we sequentially excluded hospitalizations involving patients younger than 18 years, nonelective admissions, and admissions associated with trauma, fracture, malignancy, or other nonelective indications. Index cases were further restricted to procedures performed on hospital day 0 to standardize perioperative exposure and reduce heterogeneity related to delayed surgery during admission.

Because the NRD captures readmissions only within the same calendar year, complete 90-day follow-up is not available for procedures performed late in the year. Therefore, for the 90-day readmission analyses, we included only index THA procedures performed from January 1 through September 30 of each year, ensuring a full 90-day observation window within the same NRD year. Index procedures performed between October and December were excluded from the readmission cohort to prevent incomplete follow-up.

### Outcomes

The primary outcome was all-cause 90-day readmission following elective primary THA. Readmission events were identified using NRD linkage variables and time-to-event metrics, reported as days from discharge to readmission.

Secondary outcomes were assessed among readmitted patients and included:

1. Any procedural intervention during readmission, defined by the presence of a recorded inpatient procedure code during the readmission;
2. Hip-related reoperation during readmission, defined as any hip-related inpatient procedure during readmission consistent with reoperation (including broad hip procedure families used in the analytic algorithm); and
3. True component-level revision THA during readmission, defined using component-level revision procedure patterns consistent with revision arthroplasty (component exchange/revision families), as operationalized in the prespecified coding algorithm.

Resource utilization outcomes included index hospitalization LOS and total hospital charges, as well as readmission LOS, readmission total charges, and time to readmission.

### Covariates

Patient- and hospital-level covariates were extracted from the NRD and included age, sex, calendar year, payer type, and hospital characteristics (including teaching status, bed size, and location). Comorbidity burden was captured using diagnosis-based comorbidity indicators and included major cardiometabolic and systemic conditions relevant to arthroplasty risk (eg, hypertension, dyslipidemia, diabetes, chronic kidney disease, chronic lung disease, congestive heart failure, sleep apnea, anemia, osteoporosis, and prior cardiovascular disease). Index-hospitalization complications were identified using diagnosis-based complication flags and clinically relevant postoperative diagnosis coding.

### Propensity score matching

Given substantial baseline differences between severe obesity and nonsevere obesity cohorts, we performed propensity score matching to reduce confounding. A propensity score for severe obesity status was estimated using a multivariable model including demographics (age, sex), calendar year, payer distribution, hospital characteristics, and major comorbidities (including cardiometabolic disease, renal disease, pulmonary disease, heart failure, sleep apnea, anemia, osteoporosis, and cardiovascular history). Patients were matched 1:1 using nearest-neighbor matching without replacement. Covariate balance after matching was assessed using standardized differences, and post-match diagnostics confirmed adequate balance across the included matching variables. After matching, 55,976 patients remained in the analytic cohort, including 28,798 patients with severe obesity and 27,178 matched nonseverely obese controls. Propensity score matching was performed on unweighted discharge-level data to preserve the internal validity of covariate balance. Following matching, NRD discharge-level sampling weights were reapplied to generate nationally representative weighted estimates for outcome reporting.

### Statistical analysis

Continuous variables were reported as mean ± standard deviation and compared using Student’s *t*-tests as appropriate. Categorical variables were compared using chi-square tests. Temporal trends in severe obesity prevalence across 2020-2022 were assessed using trend testing across ordered years. In the matched cohort, effect estimates for binary outcomes were summarized as odds ratios with 95% confidence intervals, derived from matched-group comparisons. All tests were 2-sided, and statistical significance was set at *P* < .05. Analyses were performed using SPSS (IBM Corp., Armonk, NY).

### Ethical aspects

The NRD contains only deidentified discharge data. This study was therefore exempt from institutional review board approval and informed consent.

## Results

### Temporal trends in severe obesity among elective primary THA

Between 2020 and 2022, a total of 366,374 weighted elective primary total hip arthroplasties met inclusion criteria. Of these, 28,798 patients (7.9%) had a diagnosis of severe obesity.

**Figure 1:**
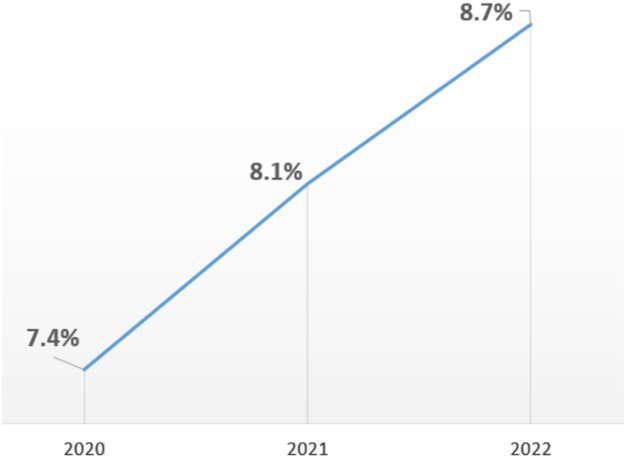
Temporal trends in severe obesity among elective primary THA, 2020-2022.

The proportion of patients with severe obesity undergoing elective primary THA increased steadily over the study period, from 7.4% in 2020 to 8.1% in 2021 and 8.7% in 2022 (*P* < .001 for trend) (Fig. 1). This represents a relative increase of approximately 17% over the 3-year period. The absolute number of severe obesity cases remained substantial across all years, reflecting the growing burden of high-risk metabolic disease within the arthroplasty population.

### Baseline characteristics before propensity score matching

Baseline demographic and clinical characteristics stratified by severe obesity status are presented in Table 1.

**Table 1:** Baseline characteristics of elective primary THA patients by severe obesity status (prematching).

Variable	Nonsevere obesity (n = 337,577)	Severe obesity (n = 28,798)	*P* value
Age, mean ± SD (y)	67.39 ± 10.81	63.05 ± 9.98	<.001
Female sex, %	56.0%	60.1%	<.001
Hypertension, %	51.4%	61.3%	<.001
Dyslipidemia, %	47.6%	51.4%	<.001
Sleep apnea, %	10.8%	32.0%	<.001
Type 2 diabetes, %	15.4%	30.5%	<.001
Chronic kidney disease, %	8.3%	11.5%	<.001
Congestive heart failure, %	1.3%	2.2%	<.001
Chronic lung disease, %	6.6%	8.8%	<.001
Mental disorders, %	31.6%	36.8%	<.001
Osteoporosis, %	5.1%	2.6%	<.001
Peripheral vascular disease, %	1.8%	1.4%	<.001
History of myocardial infarction, %	3.7%	3.8%	.535
History of cerebral vascular accident, %	4.5%	3.9%	<.001
Thyroid disorders, %	15.9%	18.1%	<.001
Index LOS, mean ± SD (d)	1.85 ± 2.11	2.36 ± 2.97	<.001
Index total charges, mean ± SD ($)	71,153 ± 46,847	75,098 ± 49,227	<.001

Patients with severe obesity were significantly younger than nonseverely obese patients (63.05 ± 9.98 vs 67.39 ± 10.81 years; *P* < .001) and more frequently female (60.1% vs 56.0%; *P* < .001).

Severe obesity was associated with a markedly higher burden of cardiometabolic comorbidities. The prevalence of type 2 diabetes was nearly doubled (30.5% vs 15.4%; *P* < .001), obstructive sleep apnea was threefold higher (32.0% vs 10.8%; *P* < .001), and hypertension was more common (61.3% vs 51.4%; *P* < .001). Chronic kidney disease (11.5% vs 8.3%), congestive heart failure (2.2% vs 1.3%), chronic lung disease (8.8% vs 6.6%), mental health disorders (36.8% vs 31.6%), and thyroid disorders (18.1% vs 15.9%) were also significantly more prevalent in the severe obesity cohort (all *P* < .001).

In contrast, osteoporosis was less common among severely obese patients (2.6% vs 5.1%; *P* < .001), consistent with their younger age distribution.

Index hospitalization resource utilization differed substantially between groups. Severe obesity was associated with longer LOS (2.36 ± 2.97 vs 1.85 ± 2.11 days; *P* < .001) and higher total hospital charges ($75,098 ± 49,227 vs $71,153 ± 46,847; *P* < .001).

These significant baseline differences justified the use of propensity score matching to reduce confounding in subsequent outcome comparisons.

### Post-propensity score matching index hospitalization outcomes

To minimize baseline differences between groups, propensity score matching was performed using nearest-neighbor matching without replacement. The matching model included age, sex, calendar year, hospital characteristics (teaching status, bed size, location), primary payer distribution, and major comorbidities including hypertension, diabetes, dyslipidemia, chronic kidney disease, chronic lung disease, congestive heart failure, sleep apnea, chronic anemia, osteoporosis, and prior cardiovascular disease. Postmatching balance diagnostics confirmed adequate covariate balance across all matched variables. After propensity score matching, 55,976 patients remained for comparison (28,798 severe obesity and 27,178 nonsevere obesity), with adequate covariate balance across matched variables.

Despite adjustment, differences in perioperative outcomes during the index hospitalization persisted between patients with and without severe obesity (Table 2).

**Table 2:** Index hospitalization outcomes after propensity score matching.

Outcome	Nonsevere obesity	Severe obesity	*P* value
Acute kidney injury (%)	2.0%	3.8%	<.001
Blood loss anemia (%)	12.8%	17.2%	<.001
Urinary tract infection (%)	0.7%	1.0%	.001
Postoperative pain diagnosis code (%)	1.3%	1.6%	<.001
Intraoperative fracture (%)	0.8%	1.0%	.014
Dislocation (%)	0.5%	0.2%	<.001
Length of stay (d), mean ± SD	1.79 ± 1.76	2.36 ± 2.97	<.001
Total charges (USD), mean ± SD	71,424 ± 46,828	75,098 ± 49,227	<.001

Severe obesity was associated with higher rates of acute kidney injury (3.8% vs 2.0%; *P* < .001), blood loss anemia (17.2% vs 12.8%; *P* < .001), urinary tract infection (1.0% vs 0.7%; *P* = .001), postoperative pain (1.6% vs 1.3%; *P* < .001), and intraoperative fracture (1.0% vs 0.8%; *P* = .014). Dislocation was slightly less frequent in the severe obesity cohort (0.2% vs 0.5%; *P* < .001).

Resource utilization remained significantly higher among patients with severe obesity, including longer LOS and greater total hospital charges.

### Resource utilization during 90-day readmission after propensity score matching

Among patients who were readmitted within 90 days, severe obesity was associated with earlier return to the hospital and greater inpatient resource utilization.

Time to readmission was shorter in patients with severe obesity (30.6 ± 24.4 days) compared with matched nonobese patients (33.9 ± 26.4 days; *P* < .001).

Readmission LOS was significantly longer in the severe obesity cohort (5.49 ± 5.34 vs 4.70 ± 4.56 days; *P* < .001). Mean readmission charges were $75,294 ± 81,212 in patients with severe obesity and $80,808 ± 102,183 in nonobese patients (*P* = .067). These findings are summarized in Table 3.

**Table 3:** Resource utilization during 90-day readmission (propensity-matched cohort).

Outcome	Nonsevere obesity	Severe obesity	*P* value
Time to readmission (d), mean ± SD	33.9 ± 26.4	30.6 ± 24.4	<.001
Readmission LOS (d), mean ± SD	4.70 ± 4.56	5.49 ± 5.34	<.001
Readmission total charges (USD), mean ± SD	80,808 ± 102,183	75,294 ± 81,212	.067

### Odds ratios for 90-day readmission and hip-related reoperations in patients with severe obesity after elective total hip arthroplasty after propensity score matching

Among matched patients, 90-day all-cause readmission occurred in 6.7% of patients with severe obesity compared with 5.8% of patients without severe obesity (*P* < .001). During readmission, any procedural intervention was performed in 5.7% of severely obese patients vs 4.0% of nonseverely obese patients (*P* < .001). Hip-related reoperation occurred in 2.4% vs 1.6%, respectively (*P* < .001), and true component-level revision surgery was required in 1.8% vs 1.2% (*P* < .001).

**Figure 2:**
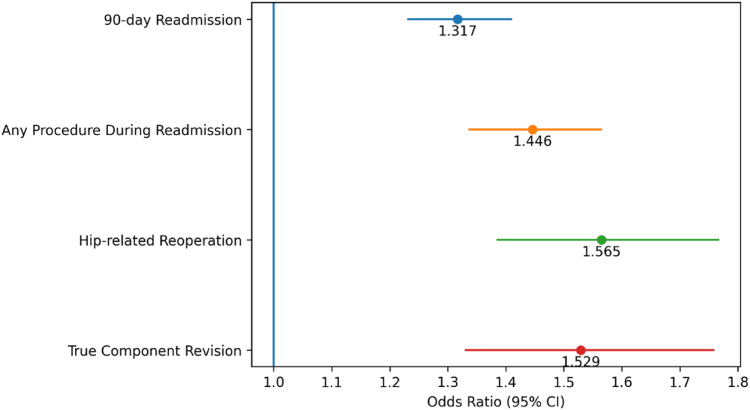
Odds ratios for 90-day readmission and hip-related reoperations in patients with severe obesity after elective total hip arthroplasty (propensity-matched cohort).

As illustrated in Figure 2, severe obesity was associated with increased odds of 90-day readmission (OR: 1.32; 95% CI: 1.23-1.41), any procedure during readmission (OR: 1.45; 95% CI: 1.34-1.56), hip-related reoperation (OR: 1.57; 95% CI: 1.39-1.77), and true component-level revision (OR: 1.53; 95% CI: 1.33-1.76).

### Causes of 90-day readmission per 10,000 index elective THA procedures after propensity score matching

When expressed per 10,000 index procedures (Table 4), severe obesity was associated with substantially higher absolute rates of infection- and wound-related readmissions. The rate of PJI was more than doubled in patients with severe obesity (65.6 vs 25.0 per 10,000). Cellulitis and respiratory complications were also markedly higher in the severe obesity cohort.

**Table 4:** Causes of 90-day readmission per 10,000 index elective THA procedures (postmatched cohort).

Cause of readmission	Nonsevere obesity (per 10,000)	Severe obesity (per 10,000)	*P* value
PJI/postop infection	25.0	65.6	<.001
Wound dehiscence	14.0	20.8	
Mechanical prosthesis issue	47.5	53.5	
Cellulitis	0.7	12.2	
Sepsis/bacteremia	46.7	44.4	
Respiratory infection/failure	5.9	22.6	
Pulmonary embolism	24.6	18.8	
Deep vein thrombosis	1.8	3.8	
Heart failure/arrhythmia	21.0	39.9	
Cute coronary syndrome/myocardial infarction/unstable angina	11.8	12.5	
Stroke/cerebrovascular	1.5	2.4	
Acute kidney injury	22.1	13.9	
Gastrointestinal complications	35.7	41.7	
Urinary tract infection/pyelonephritis	16.6	9.4	
Postop pain/hematoma	6.6	38.9	
Syncope/hypotension	23.9	4.5	
Anemia/blood loss	3.7	4.5	
Delirium/encephalopathy	0.0	4.5	

Postoperative pain/hematoma-related readmissions were significantly more frequent among patients with severe obesity (38.9 vs 6.6 per 10,000), as were heart failure and arrhythmia-related readmissions (39.9 vs 21.0 per 10,000).

In contrast, acute kidney injury and syncope-related readmissions were more frequent among patients without severe obesity.

The overall distribution of readmission diagnoses differed significantly between groups (*P* < .001).

## Discussion

In this contemporary national propensity score-matched study spanning 2020 to 2022, we found that severe obesity is both increasingly prevalent and independently associated with clinically meaningful early adverse outcomes after elective primary THA. The proportion of elective THA patients with severe obesity increased from 7.4% in 2020 to 8.7% in 2022, representing a relative rise of approximately 17% over 3 years. After matching for demographics, payer, hospital characteristics, and major comorbidities, severe obesity remained independently associated with higher index complications, longer LOS and greater charges, increased odds of 90-day readmission, and higher likelihood of procedural intervention, hip-related reoperation, and true component-level revision during readmission.

These findings are particularly relevant in the post-COVID-19 arthroplasty environment, during which patient selection, outpatient migration, perioperative optimization pathways, and surgical practice patterns have evolved substantially. As elective arthroplasty care shifted toward accelerated recovery models and selective inpatient utilization, understanding the residual impact of severe obesity on early failure risk in this modern era is increasingly important. Our study therefore provides contemporary national evidence reflecting current practice patterns rather than historical prepandemic cohorts.

Recent contemporary studies have similarly demonstrated that elevated BMI remains associated with increased perioperative burden despite advances in perioperative protocols and surgical technique [5,[12], [13], [14]]. However, many of these analyses focus primarily on complications or short-term metrics without distinguishing between readmission alone and true surgical escalation requiring reoperation or revision [12,15]. Our findings extend this literature by demonstrating that severe obesity is not only associated with readmission but also with higher odds of component-level revision within 90 days, representing clinically meaningful early implant failure rather than transient postoperative morbidity.

Several recent database studies have reported higher complication rates, increased LOS, and higher inpatient costs among obese and morbidly obese THA patients [13,[16], [17], [18]]. In particular, infection-related complications and wound issues continue to be disproportionately represented in patients with elevated BMI [19,20]. Our diagnosis-level analysis aligns with these reports and further quantifies the absolute burden per 10,000 index procedures, demonstrating more than a twofold increase in PJI-related readmissions in severely obese patients. These findings suggest that infection- and wound-related pathways remain central drivers of early failure in this population despite modern perioperative optimization strategies.

Importantly, some contemporary investigations have suggested that when surgery is performed in optimized settings or by high-volume surgeons, the impact of obesity on certain functional outcomes may be attenuated [14,21,22]. Nevertheless, even in such settings, resource utilization and perioperative complexity remain higher [5,17]. Our matched analysis, which accounted for hospital characteristics and comorbid burden, suggests that severe obesity confers residual risk beyond measured confounders, particularly along infection and wound-related pathways.

Although the absolute differences in readmission and complication rates between cohorts were numerically modest, these findings should be interpreted in the context of the very large national procedural volume of THA. Even a 1% absolute increase translates into a substantial incremental burden of early complications, reoperations, and healthcare utilization at the population level. Moreover, the observed increases in reoperation and true component-level revision represent clinically meaningful endpoints reflecting early implant failure rather than transient postoperative events, thereby supporting the clinical relevance of these differences despite small absolute percentages.

Interestingly, dislocation was less frequently coded among severely obese patients. One plausible explanation is the preferential use of stability-enhancing strategies in high-risk patients, including dual-mobility constructs and larger femoral heads, which have become increasingly utilized in contemporary practice. Additionally, the progressive shift toward anterior and laterally based surgical approaches—relative to the historically dominant posterior approach—may mitigate instability risk in selected patients. However, these modern approaches may also be technically more demanding in individuals with severe obesity due to soft tissue envelope thickness, exposure constraints, and retractor-related forces, potentially contributing to the higher rates of wound complications, infection, and blood loss anemia observed in our cohort. Because surgical approach is not captured in administrative databases, future prospective investigations are warranted to evaluate the interaction between obesity severity and approach selection in contemporary THA.

From a systems perspective, the growing prevalence of severe obesity among arthroplasty patients has important implications for bundled-payment models and value-based care initiatives. Recent cost analyses have highlighted the disproportionate contribution of high-BMI patients to episode-of-care expenditures following THA [16,18]. When coupled with our observed increase in early reoperation and revision risk, these findings emphasize the importance of targeted preoperative optimization, perioperative mitigation strategies, and closer early postoperative surveillance in this high-risk population.

Importantly, by focusing on contemporary post-COVID-19 national data with complete 90-day follow-up and by differentiating true component-level revision from general readmission events, this study provides granular insight into early failure pathways that has not been captured in prior large registry analyses of obesity in THA. This distinction strengthens the clinical relevance of our findings and addresses a key knowledge gap regarding early surgical escalation in the modern arthroplasty era.

This study has several limitations. First, the NRD is an administrative database dependent on accurate coding and lacks granular clinical variables such as body mass index values, surgical approach, implant type (eg, dual mobility constructs), operative time, blood loss, anesthesia protocols, and details of enhanced recovery pathways. As such, we cannot directly assess how modern enhanced recovery after surgery protocols, anesthetic techniques, or specific surgical approaches mediated observed outcomes. Second, the comparison group labeled “nonseverely obese” includes patients across a wide BMI spectrum (normal weight to class I obesity), which may attenuate effect estimates. Third, because the NRD captures only inpatient admissions, outpatient THA procedures—now increasingly common in the post-COVID era—are not represented, potentially biasing the cohort toward higher-risk patients selected for inpatient surgery and limiting generalizability to lower-risk ambulatory populations. Accordingly, these findings may be most generalizable to inpatient elective THA and may not extrapolate to lower-risk ambulatory THA populations. Fourth, causality cannot be inferred due to the retrospective observational design, and residual confounding may persist despite propensity matching. Finally, administrative coding does not permit differentiation of surgical approach; therefore, we cannot directly evaluate the interaction between severe obesity and contemporary anterior or lateral approaches, which have become more prevalent in recent years and may be differentially affected by obesity-related exposure challenges.

## Conclusions

Severe obesity is increasingly prevalent among patients undergoing elective primary THA and is independently associated with higher early complications, greater resource utilization, increased 90-day readmission, and elevated risk of procedural intervention, hip-related reoperation, and component-level revision during readmission. Infection- and wound-related pathways appear to account for a substantial portion of the excess readmission burden. These findings support focused preoperative optimization, targeted perioperative mitigation strategies, and enhanced early postoperative surveillance for severely obese patients to reduce early failure and resource utilization in contemporary arthroplasty practice.

## Conflicts of interest

The authors declare there are no conflicts of interest.

For full disclosure statements refer to https://doi.org/10.1016/j.artd.2026.102008.

## CRediT authorship contribution statement

**David Maman:** Writing – original draft, Methodology, Formal analysis, Data curation, Conceptualization. **Yaniv Steinfeld:** Writing – review & editing, Supervision, Conceptualization. **Yaron Berkovich:** Writing – review & editing, Supervision, Methodology.

## Appendix A . Supplementary data

Conflict of Interest Statement for Berkovich

Conflict of Interest Statement for Maman

Conflict of Interest Statement for Steinfeld
