# Supplementary material for: The Growing Impact of Severe Obesity on 90-Day Outcomes After Elective Primary Total Hip Arthroplasty: A National Propensity-Matched Study
Source: Arthroplast Today. 2026 Apr 9;39:102008. doi: 10.1016/j.artd.2026.102008 (PMC13091301; doi:10.1016/j.artd.2026.102008)
Supplement: Conflict of Interest Statement for Maman [file mmc2.docx]

# CONFLICT OF INTEREST STATEMENT

American Association of Hip and Knee Surgeons
(Adopted from the American Academy of Orthopaedic Surgeons disclosure statement)

Manuscript Title:
**The Growing Impact of Severe Obesity on 90-Day Outcomes After Elective Primary Total Hip Arthroplasty: A National Propensity-Matched Study**

1. Royalties from a company or supplier — None.

2. Speakers bureau/paid presentations for a company or supplier — None.

3A. Paid employee for a company or supplier — None.

3B. Paid consultant for a company or supplier — None.

3C. Unpaid consultant for a company or supplier — None.

4. Stock or stock options in a company or supplier — None.

5. Research support from a company or supplier as a Principal Investigator — None.

6. Other financial or material support from a company or supplier — None.

7. Royalties, financial or material support from publishers — None.

8. Medical/Orthopaedic publications editorial/governing board — None.

9. Board member/committee appointments for a society — None.

Author Name (Print or Type): David Maman, MD

Author Signature: _____________________________

Date: ___________30.01.2026__________________
